# A multicenter, randomized, double-blind, double-simulated, positive drug parallel-controlled clinical trial to evaluate the efficacy and safety of Xiao’er Fengreqing oral liquid in the treatment of acute pharyngitis/tonsillitis in children

**DOI:** 10.3389/fphar.2026.1878882

**Published:** 2026-08-21

**Authors:** Lei Shi, Kai Liu, Yike Song, Chunsun Mu, Chunxiang Wang, Yongchao Jiang, Robb Russell, Yaqian Wen, Rihe Liu, Jianping Liu, Huilan Liu, Zhaolan Liu

**Affiliations:** 1 Centre for Evidence-Based Chinese Medicine, Beijing University of Chinese Medicine, Beijing, China; 2 The Second Affiliated Hospital of Shandong University of Traditional Chinese Medicine, Jinan, Shandong, China; 3 Xiangyang Central Hospital, Xiangyang, Hubei, China; 4 Southern California University of Health Sciences, SCU Health, Whittier, CA, United States; 5 Center for Evidence-Based Medicine, Hospital of Chengdu University of Traditional Chinese Medicine, Chengdu, China; 6 Beijing University of Chinese Medicine Third Affiliated Hospital, Beijing, China

**Keywords:** acute pharyngitis, acute tonsillitis, multicenter, pediatrics, randomized controlled trial, traditional Chinese medicine, Xiao’er Fengreqing oral liquid

## Abstract

**Background:**

Acute pharyngitis and tonsillitis are common in children. Xiao’er Fengreqing Oral Liquid (XFQOL) shows potential benefits in previous studies of pediatric acute upper respiratory tract infections and influenza, but high-quality clinical evidence for pediatric acute pharyngitis/tonsillitis remains limited.

**Methods:**

This multicenter, block-randomized, double-blind, double-dummy, active-controlled trial enrolled 120 children from three tertiary hospitals. Participants were randomly assigned in a 1:1 ratio to receive either XFQOL plus Xiao’er Yanbian Granules (XYG) placebo (XFQOL group, n = 60) or XYG plus XFQOL placebo (control group, n = 60) for 5 days. Primary efficacy endpoints were the day-5 response and disappearance rates of sore throat, assessed using the Wong-Baker Faces Pain Rating Scale (WBS). Secondary endpoints included the time to WBS response and disappearance, the day-3 WBS response rate, and changes in traditional Chinese medicine (TCM) syndrome scores and individual symptoms and signs. Safety was assessed via adverse events and adverse drug reactions.

**Results:**

A total of 120 participants were included in the Full Analysis Set. Baseline characteristics were comparable between the groups. Response rate for the WBS sore throat score after 5 days of treatment was 100% in both groups. The disappearance rate was 98.33% in the experimental group and 90% in the control group, with a hazard ratio and its 95% confidence interval (experimental group/control group) of 0.0833 (97.5% CI: 0.0008–0.1659). Non-inferiority testing demonstrated that the experimental group was not inferior to the control group. In addition, statistically significant intergroup differences were observed in TCM syndrome sore throat onset time and TCM syndrome sore throat disappearance time, no statistically significant differences were detected in any other indicators. No related adverse events were attributed to XFQOL.

**Conclusion:**

XFQOL is non-inferior to XYG for the 5-day sore throat disappearance rate in pediatric acute pharyngitis/tonsillitis. Secondary endpoint analyses suggest potential advantages in the speed of symptom relief, including a shorter time to sore throat disappearance and a higher 3-day response rate, while the effects on TCM syndrome total scores and most individual symptom/sign scores are comparable between groups. Furthermore, XFQOL demonstrates a favorable safety profile, with no treatment-related adverse events identified, suggesting its potential for clinical application.

**Clinical Trial Registration:**

clinicaltrials.gov, ITMCTR2024000569.

## 1 Introduction

Acute pharyngitis and acute tonsillitis are among the most common upper respiratory tract infectious diseases in children. The main clinical symptoms are pharyngeal discomfort, pain, or odynophagia, with local manifestations including swelling of the lymphoid follicles on the posterior pharyngeal wall and congestion of the palatine tonsils (Góra and Michałek-Piernik, 2024; Borschmann and Berkowitz, 2006; ICD-11 for Mortality and Morbidity Statistics). Most cases of acute pharyngitis/tonsillitis in children are caused by viral infections, including adenovirus, rhinovirus, influenza virus, enterovirus, Epstein–Barr virus, and severe acute respiratory syndrome coronavirus 2. Group A β-hemolytic *Streptococcus* is the most common bacterial cause and accounts for approximately 20%–30% of acute pharyngitis cases in children. Less common bacterial pathogens include group C and G streptococci, *Arcanobacterium haemolyticum*, *Corynebacterium diphtheriae*, and *Neisseria gonorrhoeae*. Viral–bacterial coinfections may also occur (Wang and Shen, 2022). At present, the clinical treatment of acute pharyngitis/tonsillitis includes local treatment and systemic treatment. Local treatment includes the use of sprays, lozenges and gargling with analgesic, antiviral and antibacterial products. Systemic treatment includes the use of antiviral and antibacterial drugs and various approaches of traditional Chinese medicine (TCM) (Hu et al., 2023). Evertheless, antibiotics continue to be frequently prescribed for acute pharyngitis in clinical practice, even though most cases are viral and self-limiting. Inappropriate or excessive antibiotic use provides limited clinical benefit and contributes to antimicrobial resistance, avoidable adverse reactions, and increased healthcare costs (Qi et al., 2023). Therefore, exploring alternative or adjunctive treatment methods that can effectively relieve symptoms with high safety is crucial.

In TCM, acute pharyngitis and acute tonsillitis are generally classified under the categories of “acute throat obstruction” (*Ji Hou Bi*) and “acute tonsillar swelling” (*Ji Ru E*), respectively. The term *Ji Hou Bi* refers to an acute disorder characterized primarily by redness, swelling, and pain in the throat, often accompanied by difficulty swallowing, and should not be equated with the Western medical concept of acute laryngitis (Wang X. et al., 2024; China Association of Chinese Medicine, 2012). The commonly recognized acute syndrome patterns include external contraction of wind-heat and exuberant heat in the lung and stomach (Wang X. et al., 2024). In recent years, Chinese herbal therapies have been evaluated in randomized trials and evidence syntheses for sore throat, acute pharyngitis/tonsillitis, and pediatric acute upper respiratory tract infections.

Xiao’er Fengreqing Oral Liquid (XFQOL) is a pure Chinese herbal preparation based on the modifications of the classic formula Yinqiao Powder (National Drug Approval Number Z19990012). This medication consists of 20 herbs: Honeysuckle Flower (Jinyinhua), Forsythia Fruit (Lianqiao), Isatis Root (Banlangen), Peppermint (Bohe), Bupleurum Root (Chaihu), Lophatherum Herb (Danzhuye), Great Burdock Achene (Niubangzi), Platycodon Root (Jiegeng), Baical Skullcap Root (Huangqin), Gardenia Fruit (Zhizi), Reed Rhizome (Lugen), Gypsum (Shigao), Schizonepeta Spike (Jingjiesui), Red Peony Root (Chishao), Bitter Apricot Seed (Kuxingren), Bitter Orange (Zhiqiao), Medicated Leaven (Liushenqu), Stiff Silkworm (Jiangcan), Saposhnikovia Root (Fangfeng), and Licorice Root (Gancao). It has the effects of dispelling exterior syndromes with pungent-cool herbs, clearing heat and detoxifying, relieving cough and soothing the throat. Previous clinical evidence has suggested the potential efficacy and safety of XFQOL in pediatric influenza and acute upper respiratory tract infection (Zhou et al., 2020; Guo et al., 2025). However, specific evidence for pediatric acute pharyngitis and tonsillitis remains limited. This study aimed to evaluate the efficacy and safety of XFQOL in the treatment of acute pharyngitis/tonsillitis (external wind-heat syndrome) in children through a multicenter, randomized, double-blind, double-simulated, positive drug parallel-controlled clinical trial.

## 2 Methods

### 2.1 Study design

This multicenter, randomized, double-blind, double-simulated, positive-controlled trial was conducted at three Chinese tertiary hospitals. Led by the Third Affiliated Hospital of Beijing University of Chinese Medicine, the Second Affiliated Hospital of Shandong University of Traditional Chinese Medicine and Xiangyang Central Hospital participated as co-investigators. This study was approved by the Scientific Research Ethics Committee of the Third Affiliated Hospital of Beijing University of Chinese Medicine on 3 September 2024, and was registered in International Traditional Medicine Clinical Trial Registry with the registry number ITMCTR2024000569 (url: https://itmctr.ccebtcm.org.cn/mgt/project/view/5193351313809723719). The protocol and statistical analysis plan have been published in Frontiers in Pharmacology.

Xiao’er Yanbian Granules was selected as the active comparator because it is an approved Chinese patent medicine included in the Chinese Pharmacopoeia, with labeled indications covering acute pharyngitis/tonsillitis and a wind-heat syndrome pattern comparable to that of XFQOL. Its clinical indication, target population, oral route of administration, and syndrome applicability are consistent with the objectives of this study.

### 2.2 Participants

#### 2.2.1 Diagnostic criteria

The diagnostic criteria were formulated with reference to “Zhu Futang Practical Pediatrics” (Wang and Shen, 2022), “Clinical Practice Guideline for the Diagnosis and Treatment of Acute Tonsillitis in Children (2016)” (Liu and Gu, 2017), “Technical Guidelines for the Design and Evaluation of Clinical Trials of Traditional Chinese Medicine for Common Pediatric Diseases Part 13: Acute Pharyngitis and Tonsillitis” (Technical Guidelines for the Design), and “Guiding Principles for Clinical Research of New Chinese Medicines (2002 Edition)” (China Association of Chinese Medicine, 2012).

The TCM pattern differentiation criteria were formulated with reference to the Chinese Association of Chinese Medicine’s “Technical Guidelines for the Design and Evaluation of Clinical Trials of Traditional Chinese Medicine for Common Pediatric Diseases Part 13: Acute Pharyngitis and Tonsillitis” (Technical Guidelines for the Design) and “Guiding Principles for Clinical Research of New Chinese Medicines (2002 Edition)” (China Association of Chinese Medicine, 2012).

During screening, investigators assessed the probable etiology of acute pharyngitis/tonsillitis based on medical history, clinical manifestations, physical examination, and laboratory markers. Children with findings suggesting bacterial infection requiring antibiotic treatment were not enrolled. Specifically, eligible children had to have a maximum axillary temperature <38.5 °C at the visit and, within 48 h before the visit, a white blood cell count, neutrophil percentage, and C-reactive protein level not exceeding the upper limits of the local normal reference ranges. Children with suppurative tonsillitis or complications requiring antibacterial treatment were excluded.

#### 2.2.2 Inclusion criteria

1. Meeting the Western medicine diagnostic criteria for acute pharyngitis and/or acute tonsillitis in children;
2. Meeting the TCM diagnostic criteria for pediatric acute tonsillitis (Ji Ru E) and/or acute laryngitis (Ji Hou Bi), pattern of external contraction of wind-heat;
3. Duration of illness ≤48 h, and baseline WBS sore throat score ≥4 points;
4. Maximum axillary temperature at the time of visit <38.5 °C;
5. Within 48 h before the visit, laboratory test reports showed white blood cell count ≤ ULN (upper limit of normal reference range); neutrophil percentage ≤ ULN; C-reactive protein ≤ ULN;
6. Age between 3 and 14 years (including 3 and 14 years old);
7. The informed consent process complied with regulations. The legal guardian was required to sign the informed consent form, and participating children aged 8 years or older were also required to provide assent/signature in addition to guardian consent.

#### 2.2.3 Exclusion criteria

1. Pharyngeal symptoms or inflammation caused by diseases such as measles, scarlet fever, infectious mononucleocytosis, agranulocytosis, diphtheria, suppurative tonsillitis, herpangina, or pharyngoconjunctival fever;
2. Children with complications (such as infectious laryngitis, bacterial otitis media, acute bronchitis, pneumonia, etc.);
3. Underlying respiratory, cardiac, renal, hepatic, hematological, endocrine, neurological, or immunodeficiency diseases judged by the investigator, based on clinical assessment and relevant laboratory tests or examinations, to make the child unsuitable for participation
4. Glucose-6-phosphate dehydrogenase deficiency (favism);
5. Known allergy to any component of XFQOL, Xiao’er Yanbian Granules, or any excipients, flavoring agents, or preservatives used in the double-simulated placebo formulations;
6. History of febrile seizures;
7. Diarrhea occurring within 48 h before the visit.

Patients will also be excluded from the study if they are unable to take oral medication, such as severe dysphagia or frequent vomiting, or if they have participated in another interventional clinical trial within 30 days prior to screening or within five half-lives of the investigational product, whichever is longer.

#### 2.2.4 Discontinuation criteria

1. Occurrence of allergic reactions or serious adverse events, where the physician judges that medication should be stopped;
2. Worsening of the condition after medication, or occurrence of complications (such as infectious laryngitis, bacterial otitis media, acute bronchitis, etc.), or occurrence of other diseases, and the investigator assesses that the child is unsuitable to continue the trial;
3. Serious violation of inclusion or exclusion criteria discovered after randomization;
4. For any reason, the child or their legal guardian is unwilling or unable to continue the clinical trial and requests withdrawal from the trial from the responsible physician;
5. The subject does not explicitly request withdrawal but no longer takes the investigational drug or undergoes testing.
6. Emergency unblinding for safety or medical management reasons.

#### 2.2.5 Elimination criteria

1. Those who were mistakenly enrolled despite not meeting the inclusion criteria or who met any of the exclusion criteria;
2. Those who, although meeting the inclusion criteria, never took the study drug after enrollment, or had no follow-up records;
3. Exclusion criteria also included documenting the self-administered use of prohibited or non-permitted concomitant medications, particularly potent influencers of the investigational drug like Prednisone Acetate Tablets or Dexamethasone, that confounded the assessment of efficacy and safety.

#### 2.2.6 Sample size estimation

Referring to relevant literature and previous clinical trials, this study used a non-inferiority design with a 1:1 group sample size allocation, α = 0.025 (one-sided), β = 0.2. The estimated disappearance rate for sore throat in the intervention group was 91.43%, and 76.92% in the control group, with a non-inferiority margin of 0.1. The calculated sample size required per group was 34 cases, totaling 68 cases. Considering a 20% dropout rate, the actual sample size per group was n = 44, totaling 88 cases. Considering that the subjects are children with potentially poorer compliance management, to increase testing power, provide more reliable statistical analysis results, and ensure the scientific reliability of the research results, the sample size was set at 120 cases.

### 2.3 Randomization, allocation concealment, and blinding

This multicenter trial employed central block randomization (block size = 4) to ensure balanced allocation between the two groups, each comprising 60 participants. An independent statistician who was not involved in recruitment, treatment, outcome assessment, or data analysis generated the randomization sequence using SAS software. Site investigators screened and enrolled eligible participants. After enrollment, a designated drug administrator at each center assigned the next sequentially numbered medication package according to the concealed allocation sequence. Allocation concealment was maintained by sequentially numbered, sealed, opaque envelopes and by identically appearing active drugs and matching placebos. To preserve blinding, medications were pre-packaged by non-trial personnel. Participants, legal guardians, investigators, outcome assessors, and data analysts remained blinded to treatment assignments throughout the study to minimize evaluation bias. All personnel received standardized training for protocol adherence.

### 2.4 Investigators and researchers

All participating researchers held senior clinical qualifications, possessed trial expertise, and passed formal reviews. Standardized training was implemented pre-trial to ensure full protocol and indicator comprehension.

### 2.5 Interventions

1. XFQOL and its simulated placebo were administered according to the following dosage: 3–6 years old, 5–10 mL/dose; 6–14 years old, 7.5–15 mL/dose; four times a day, shake well before use.
2. Xiao’er Yanbian Granules and its simulated placebo were administered according to the following dosage: orally, 3–5 years old, one sachet (4 g) each time, three times a day; 6–14 years old, two sachets (8 g) each time, 2–3 times a day.
3. The experimental group received XFQOL and the Xiao’er Yanbian Granules placebo; the control group received Xiao’er Yanbian Granules and the XFQOL placebo. The treatment course for both groups was 5 days, with a telephone follow-up 24 h after treatment termination.

### 2.6 Concomitant medications

1. All medications used before enrollment and during the trial were documented in the case report forms and source medical records, including their generic names, dates and times of administration, dosages, and reasons for use. During the observation period, the use of antibiotics, antiviral agents, Chinese herbal medicines with similar indications, and any other medications or interventions that could potentially affect the treatment outcomes of acute pharyngitis/tonsillitis was prohibited, except for the assigned investigational treatments;
2. If the patient’s body temperature (axillary) was ≥38.2 °C, to protect the subject, the investigator could add acetaminophen as needed. The dosage of acetaminophen followed the instructions. However, if persistent high fever did not subside, prompt medical attention was required;
3. All concomitant medications for subjects were recorded in the case report form and original medical records, including the generic name, administration time, dosage, and reason for use.

### 2.7 Endpoints

#### 2.7.1 Primary endpoints

The primary efficacy endpoints were the response rate and disappearance rate of the WBS sore throat score after 5 days of medication.

#### 2.7.2 Secondary endpoints

The secondary efficacy endpoints were the time to onset and time to disappearance of the WBS sore throat score; the response rate and disappearance rate of the WBS sore throat score after 3 days of medication; time to complete fever resolution; the response rate and disappearance rate based on the total TCM syndrome score after 5 days; the time to onset and time to disappearance of the TCM syndrome item “sore throat”; the response rate and disappearance rate for the TCM syndrome item “sore throat” after 3 and 5 days of medication; and the response rate and disappearance rate for individual TCM symptoms/signs scores after 5 days of medication.

### 2.8 Safety analysis

Observation indicators: (1) Incidence of clinical adverse events (AEs)/adverse drug reactions (ADRs); (2) Vital signs; (3) Laboratory tests: complete blood count, C-reactive protein, urinalysis, liver function, kidney function and electrocardiogram. The incidence of adverse reactions was the primary safety indicator.

### 2.9 Statistical methods

SAS 9.4 software was used for statistical analysis. For the primary indicators, the confidence interval method was used for analysis. Efficacy analyses were primarily performed in the full analysis set (FAS) according to the intention-to-treat principle, and supportive analyses were performed in the per-protocol set (PPS). Safety analyses were performed in the safety set (SS). Because all randomized participants completed the trial without major protocol violations, the FAS, PPS, and SS populations were identical in this study. If the lower limit of the confidence interval for the difference in effect size between the experimental group and the control group was greater than the negative non-inferiority margin, non-inferiority was considered established. For secondary indicators, two-sided tests were used, and a P ≤ 0.05 was considered statistically significant. For numerical outcome data, data were described by the number of cases, mean, standard deviation, minimum, median, maximum, upper quartile (Q1), lower quartile (Q3), and 95% confidence interval (95% CI, using a one-sided 97.5% CI for the primary outcome indicators). Inter-group comparisons were performed using t-tests or Wilcoxon rank-sum tests. For categorical outcome indicator data, data were described by frequency tables, percentages, or constituent ratios. For inter-group comparisons, chi-square tests, Fisher’s exact test were used. If considering the influence of center or other factors, CMH chi-square test or logistic regression analysis were used. Missing values in effectiveness endpoints would use the LOCF method for the FAS. LOCF assumes that the last observed value remains unchanged for subsequent missing timepoints, providing a conservative estimate of treatment effects. For safety endpoints, no imputation is applied; analyses rely solely on observed data to avoid overestimating or underestimating adverse event rates.

## 3 Results

### 3.1 Trial completion and dataset division

**FIGURE 1:**
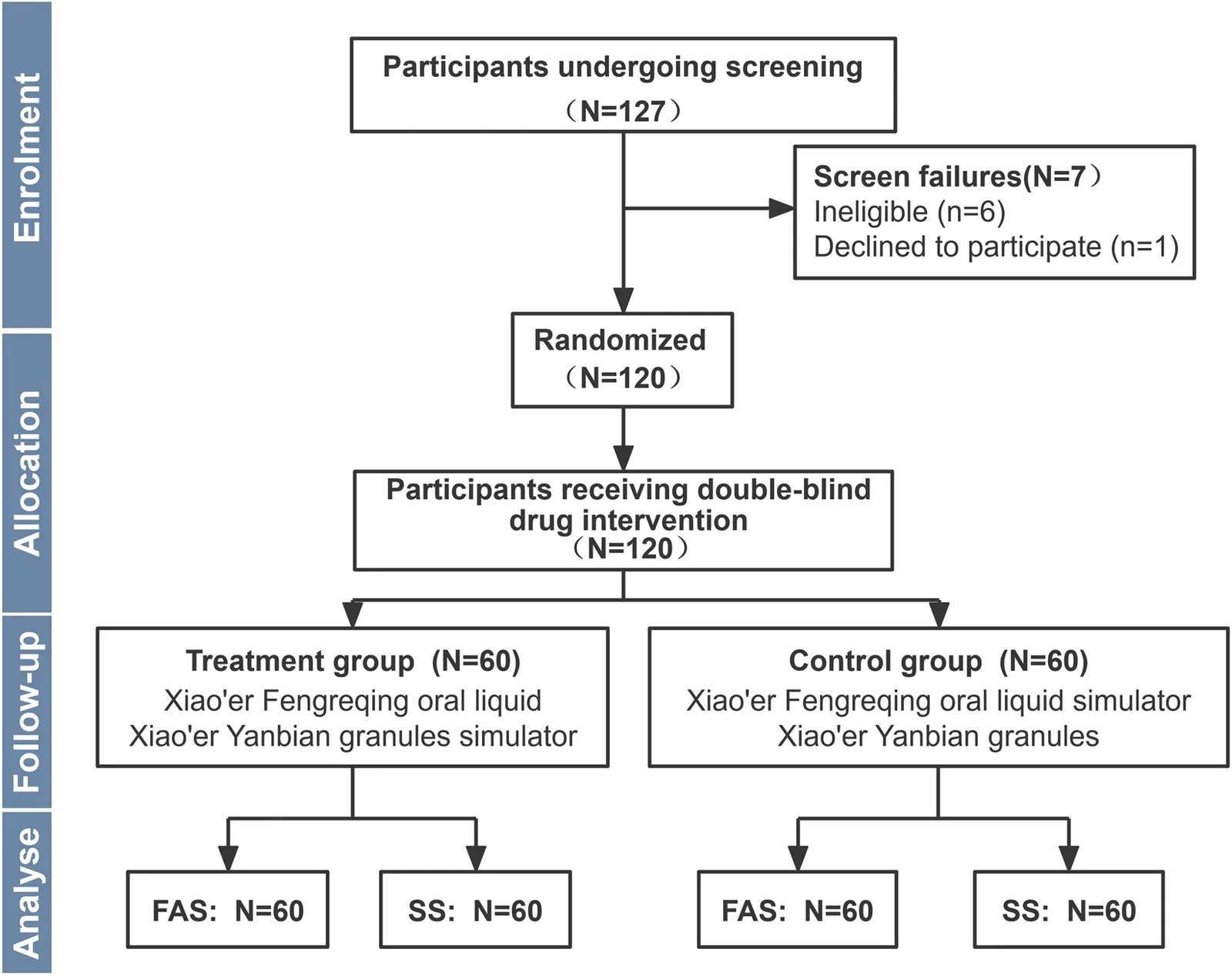
Completion of the trial and dataset division. Screened: n = 127; excluded before randomization: n = 7; randomized: n = 120; allocated to experimental group: n = 60; allocated to control group: n = 60; lost to follow-up: n = 0; discontinued intervention: n = 0; discontinued because of adverse events: n = 0; analyzed in FAS/PPS/SS: n = 60 per group. SS, safety set; FAS, full analysis set; PPS, per-protocol set.

This trial started in October 2024 and was completed in March 2025. A total of 127 children were screened, among whom seven were excluded prior to randomization. Reasons for screening exclusion included refusal to participate (n = 1), meeting exclusion criterion 6 (n = 1), and not meeting inclusion criterion 5 (n = 5). A total of 120 enrolled children were randomly assigned to the experimental or control group in a 1:1 ratio. The FAS included 120 subjects; the SS included 120 subjects. Detailed procedures are illustrated in Figure 1.

### 3.2 Distribution of subjects

A total of 120 subjects were enrolled in this study across three research centers, including 60 in the experimental group and 60 in the control group, with no dropouts or exclusions (Table 1; Supplementary Table S1). The subjects included in the FAS set, PPS set, and SS set were the same, with 60 in the experimental group and 60 in the control group in each set (Table 2), all patients completed the trial without taking any prohibited drugs.

**TABLE 1:** The completion status of experiments at each research center.

Center name	Control group		Experimental group		Total	
	Selected	Remove/fall off	Selected	Remove/fall off	Selected	Remove/fall off
Beijing University of Chinese Medicine Third Affiliated Hospital	2	0	2	0	4	0
The Second Affiliated Hospital of Shandong University of Traditional Chinese Medicine	40	0	40	0	80	0
Xiangyang Central Hospital	18	0	18	0	36	0

**TABLE 2:** Distribution of subjects.

Group	Selected	Fall off	FAS	PPS	SS
Control group	60	0	60	60	60
Experimental group	60	0	60	60	60
Total	120	0	120	120	120

### 3.3 Baseline characteristics

There were no statistically significant differences between the two groups in demographic data (sex, ethnicity, age, height, weight), disease conditions (duration of illness, body temperature, maximum axillary temperature within 24 h before diagnosis, family history, allergy history), disease diagnosis (WBS facial expression quantitative score, acute pharyngitis, acute tonsillitis, external contraction of wind-heat syndrome), or laboratory test indicators (white blood cell count WBC, neutrophil percentage NEUT%) (Table 3). Efficacy-related indicators, including TCM syndrome scores, individual symptom scores, and symptom dimension scores, showed no statistically significant differences between groups, indicating comparability.

**TABLE 3:** Comparison of baseline data between the two groups.

Variable	Description	Control group (n = 60)	Experimental group (n = 60)	Z/χ^2^/t value	P -value
Demographics					
Gender (n)	Male	30	30	0.000^a^	1.000
	Female	30	30	—	—
Ethnicity (n)	Han	59	60	—^b^	1.000
	Other	1	0	—	—
Age (years)	Mean (SD)	6.1 (2.78)	6.2 (2.56)	−0.680^a^	0.496
Height (cm)	Median (Q1–Q3)	111 (104, 119.5)	115 (106, 124)	−0.882^c^	0.378
Weight (Kg)	Median (Q1–Q3)	19.5 (17, 26.25)	21 (17.5, 25)	−0.505^c^	0.614
Disease conditions					
Disease course (h)	Median (Q1–Q3)	23 (10, 24)	24 (6.5, 31)	−1.212^c^	0.226
Temperature (°C)	Median (Q1–Q3)	37 (36.6, 37.35)	37.1 (36.6, 37.5)	−0.574^c^	0.566
Max axillary temp in 24 h pre-visit (°C)	Median (Q1–Q3)	37.5 (36.7, 37.9)	37.65 (36.7, 38)	−0.671^c^	0.503
Allergy history	No	58 (96.7)	58 (96.7)	0.000^d^	1.000
	Yes	2 (3.3)	2 (3.3)	—	—
Family history	No	60 (100)	60 (100)	—	—
	Yes	0 (0)	0 (0)	—	—
Disease diagnosis					
WBS face scale score	Median (Q1–Q3)	6 (6, 6)	6 (6, 7)	−0.021^c^	0.983
Acute pharyngitis	Yes	35 (58.3)	40 (66.7)	0.889^b^	0.346
	No	25 (41.7)	20 (33.3)	—	—
Acute tonsillitis	Yes	33 (55.0)	28 (46.7)	0.834^b^	0.361
	No	27 (45.0)	32 (53.3)	—	—
Wind-heat syndrome	Yes	60 (100)	60 (100)	—	—
	No	0 (0)	0 (0)	—	—
Laboratory tests					
WBC (10^9/L)	Mean (SD)	6.4 (1.25)	6.4 (1.32)	−0.052^a^	0.958
NEUT%	Mean (SD)	52.7 (8.35)	55.1 (8.03)	−1.614^a^	0.109

^a^ Independent samples t-test.

^b^ χ2 test Pearson formula.

^c^ Wilcoxon rank-sum test.

^d^ χ2 test Continuity correction.

### 3.4 Primary evaluation indicators

#### 3.4.1 Comparison of WBS sore throat response rate after 5 days of medication

After treatment, the WBS sore throat response rate, assessed using the Wong-Baker FACES Pain Rating Scale, was 100% (60/60) in both the control and experimental groups. The observed risk difference was 0. Because there was no variation in the rates between the two groups, the standard error of the risk difference was 0, and its one-sided 97.5% confidence interval was [0, 0] (Table 4). According to the preset non-inferiority margin of 0.1, the lower limit of the confidence interval was 0, satisfying condition 0 > −0.1 (Table 5). Therefore, the conclusion that the control group is non-inferior to the experimental group in terms of response rate holds true. Similarly, the conclusion that the experimental group is non-inferior to the control group also holds true.

**TABLE 4:** Fourfold table for WBS sore throat response rate after 5 days of medication.

Primary endpoint	Description	Control group (n = 60)	Experimental group (n = 60)	Total (n = 120)
WBS sore throat response rate after 5 days	Response	60	60	120
	No response	0	0	0

**TABLE 5:** Statistical table for WBS sore throat response rate after 5 days of medication.

Response rate	Risk difference	ASE (standard error)	Non-inferiority test	One-sided 97.5% confidence interval
Comparison (control group - experimental group)	0.0	0.0	0.0 > −0.1	0.0, 0.0

#### 3.4.2 Comparison of WBS sore throat disappearance rate after 5 days of medication

**FIGURE 2:**
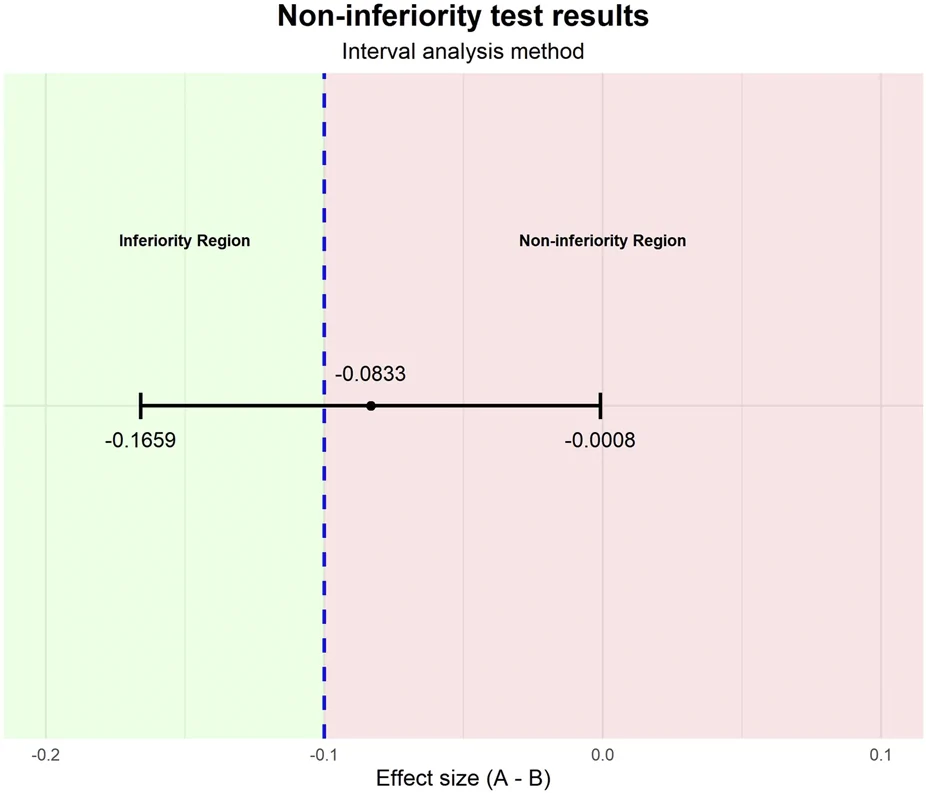
Non-inferiority test plot (control group vs. experimental group).

**FIGURE 3:**
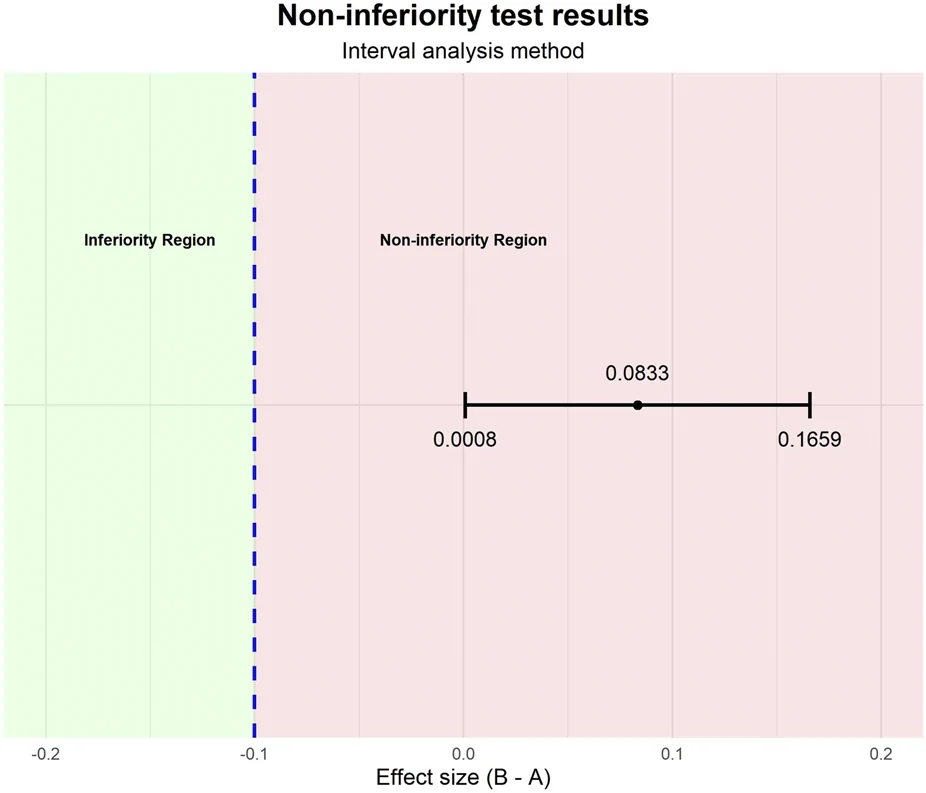
Non-inferiority test plot (experimental group vs. control group).

For the disappearance rate endpoint, the disappearance rate was 90% (54/60) in the control group and 98.33% (59/60) in the experimental group (Table 6). As shown in Figure 2 (risk difference analysis: control - experimental), the observed risk difference (control - experimental) was −0.0833, with a one-sided 97.5% confidence interval of [−0.1659, −0.0008]. The lower limit of the confidence interval was −0.1659. According to the preset non-inferiority margin of 0.1, the condition of being greater than −0.1 was not met (Table 7). Therefore, the conclusion that the control group is non-inferior to the experimental group in terms of disappearance rate is not established. Since the data analysis was blinded, the risk difference (experimental - control) was also calculated as 0.0833, with a one-sided 97.5% confidence interval of [0.0008, 0.1659]. The lower limit of this interval was 0.0008, satisfying the condition of being greater than −0.1, as shown in Figure 3 (risk difference analysis: experimental - control). Therefore, the conclusion that the experimental group is non-inferior to the control group in terms of disappearance rate is established.

**TABLE 6:** Fourfold table for WBS sore throat disappearance rate after 5 days of medication.

Primary endpoint	Description	Control group (n = 60)	Experimental group (n = 60)	Total (n = 120)
WBS sore throat disappearance rate after 5 days	Disappeared	54	59	113
	Not disappeared	6	1	7

**TABLE 7:** Statistical table for WBS sore throat disappearance rate after 5 days of medication.

Disappearance rate comparison	Risk difference	ASE (standard error)	Non-inferiority test	One-sided 97.5% CI	Non-inferiority established
Comparison (control group - experimental group)	−0.0833	0.0421	−0.1659 < −0.1000	−0.1659, −0.0008	No
Comparison (experimental group - control group)	0.0833	0.0421	0.0008 > −0.1000	0.0008, 0.1659	Yes

### 3.5 Secondary evaluation indicators

The median time to complete fever resolution was 11 h in the experimental group and 11 h in the control group, with no statistically significant difference between groups by Wilcoxon rank-sum test (P = 0.759). There was no statistically significant difference in the time to onset of WBS sore throat between groups (P = 0.412), but there was a statistically significant difference in the time to disappearance of WBS sore throat between groups (P = 0.014). There was a statistically significant difference in the WBS sore throat response rate after 3 days of medication between groups (P = 0.002), but no statistically significant difference in the WBS sore throat disappearance rate after 3 days (P = 0.178). There were no statistically significant differences between groups in the response rate and disappearance rate based on the total TCM syndrome score after 5 days. There were statistically significant differences between groups in the time to onset and time to disappearance of the TCM syndrome item “sore throat” (P < 0.05). There were no statistically significant differences between groups in the response rate and disappearance rate for individual TCM symptoms/signs scores after 5 days of medication (Table 8). Because no formal multiplicity adjustment was applied to secondary endpoints, these P values should be interpreted as nominal, and the secondary findings should be regarded as supportive and exploratory/hypothesis-generating.

**TABLE 8:** Comparison of secondary endpoints between groups.

Secondary endpoints	Description	Control group (n = 60)	Experimental Group (n = 60)	Z/χ2/t value	P value
Time to complete defervescence (h)	Median (Q1–Q3)	11 (10, 12)	11 (9, 12)	0.307^a^	0.759
WBS sore throat score					
WBS sore throat onset time (days)	Median (Q1–Q3)	1 (1, 2)	1 (1, 2)	0.820^a^	0.412
WBS sore throat disappearance time (days)	Median (Q1–Q3)	4 (3.5)	4 (3, 4)	2.467^a^	0.014
WBS sore throat response rate after 3 days	Response	45 (75.0)	57 (95.0)	9.412^b^	0.002
	No response	15 (25.0)	3 (5.0)	—	—
WBS sore throat disappearance rate after 3 days	Disappeared	17 (28.3)	24 (40.0)	1.815^b^	0.178
	Not disappeared	43 (71.7)	36 (60.0)	—	—
TCM syndrome efficacy					
TCM syndrome total score response rate after 5 days	Response	59 (98.3)	60 (100.0)	–^c^	1.000
	No response	1 (1.7)	0 (0.0)	—	—
TCM syndrome total score disappearance rate after 5 days	Disappeared	51 (85.0)	55 (91.7)	1.294^b^	0.255
	Not disappeared	9 (15.0)	5 (8.3)	—	—
TCM syndrome sore throat onset time (days)	Median (Q1–Q3)	2 (1, 3)	2 (1, 2)	2.839^a^	0.005
TCM syndrome sore throat disappearance time (days)	Median (Q1–Q3)	4 (3, 5)	4 (3, 4)	2.325^a^	0.020
Response/Disappearance rate of individual TCM symptoms/signs after 5 days					
Main symptom					
Sore throat (dysphagia) response rate	Response	58 (96.7)	60 (100.0)	0.508^d^	0.476
	No response	2 (3.3)	0 (0.0)	—	—
Sore throat (dysphagia) disappearance rate	Disappeared	57 (95.0)	59 (98.3)	0.259^d^	0.611
	Not disappeared	3 (5.0)	1 (1.7)	—	—
Secondary symptoms					
Dry throat discomfort response rate	Response	59 (98.3)	60 (100.0)	–^c^	1.000
	No response	1 (1.7)	0 (0.0)	—	—
Dry throat discomfort disappearance rate	Disappeared	57 (95.0)	57 (95.0)	—	—
	Not disappeared	3 (5.0)	3 (5.0)	0.000^d^	1.000
Fever response rate	Response	60 (100)	60 (100)	—	—
	No response	0 (0)	0 (0)	—	—
Fever disappearance rate	Disappeared	60 (100)	60 (100)	—	—
	Not disappeared	0 (0)	0 (0)	—	—
Cough with yellow sputum response rate	Response	57 (95.0)	59 (98.3)	0.259^d^	0.611
	No response	3 (5.0)	1 (1.7)	—	—
Cough with yellow sputum disappearance rate	Disappeared	57 (95.0)	59 (98.3)	0.259^d^	0.611
​	Not disappeared	3 (5.0)	1 (1.7)	—	—
Signs					
Pharyngeal signs response rate	Response	58 (96.7)	60 (100.0)	0.508^d^	0.476
	No response	2 (3.3)	0 (0.0)	—	—
Pharyngeal signs disappearance rate	Disappeared	57 (95.0)	60 (100.0)	1.368^d^	0.242
	Not disappeared	3 (5.0)	0 (0.0)	—	—
Tonsillar signs response rate	Response	59 (98.3)	59 (98.3)	0.000^d^	1.000
	No response	1 (1.7)	1 (1.7)	—	—
Tonsillar signs disappearance rate	Disappeared	56 (93.3)	59 (98.3)	0.835^d^	0.361
	Not disappeared	4 (6.7)	1 (1.7)	—	—
Lymphadenopathy response rate	Response	59 (98.3)	60 (100.0)	-^c^	1.000
	No response	1 (1.7)	0 (0.0)	—	—
Lymphadenopathy disappearance rate	Response	59 (98.3)	60 (100.0)	–^c^	1.000
	No response	1 (1.7)	—	—	—

^a^ Wilcoxon rank-sum test.

^b^ χ2 test Pearson formula.

^c^ χ2 test Fisher’s Exact.

^d^ χ2 test Continuity correction.

### 3.6 Safety analysis

A total of four adverse events occurred during the trial, all presenting as abnormal laboratory test indicators (urine protein positive). Among them, 1 case occurred in the experimental group and 3 cases in the control group. The investigators judged the relationship to the drug as “possibly unrelated”. Medication was continued, no measures were taken, and the outcomes were all “resolved”. There was no significant difference in the incidence of adverse events between the experimental group and the control group (Table 9). No adverse reactions were observed during the study, and safety was good. The detailed occurrence and outcome of adverse events are presented in Supplementary Table S1, the laboratory safety inspection and physical safety inspection indicators are shown in Supplementary Tables S2,S3, respectively, and there is no statistically significant difference between the two groups.

**TABLE 9:** Comparison of adverse event incidence between the two groups (SS).

Adverse event	Control group (n = 60)	Experimental group (n = 60)	Total (n = 120)	Statistic	P value
N (missing)	60	60	120	1.034	0.309
Occurred	3	1	4	—	—
Not occurred	57	59	116	—	—

## 4 Discussion

Acute pharyngitis and tonsillitis are among the most common upper respiratory tract infections in children and are frequent reasons for pediatric outpatient visits. Although most uncomplicated cases are self-limiting and are commonly associated with viral infection, sore throat, odynophagia, fever, and local pharyngeal inflammation can substantially affect eating, sleep, daily activities, and the concerns of caregivers. At the same time, the inappropriate use of antibiotics for non-bacterial upper respiratory tract infections contributes to antimicrobial resistance and avoidable treatment burden (Antimicrobial Resistance Collaborators, 2022; Spinks et al., 2021). Therefore, effective and safe non-antibiotic approaches that can rapidly relieve symptoms are clinically valuable.

In this multicenter, randomized, double-blind, double-simulated, active-controlled trial, Xiao’er Fengreqing Oral Liquid (XFQOL) was evaluated in children with acute pharyngitis/tonsillitis characterized by an external wind-heat syndrome pattern. The principal finding was that XFQOL was non-inferior to Xiao’er Yanbian Granules (XYG) with respect to the 5-day disappearance rate of sore throat. The 5-day WBS response rate reached 100% in both groups, while the disappearance rate was 98.33% in the XFQOL group and 90.00% in the XYG group. In addition, secondary endpoint analyses showed a shorter time to disappearance of sore throat and a higher day-3 response rate in the XFQOL group. Taken together, these results indicate that XFQOL provides overall symptom control comparable to that of the active comparator and may offer an advantage in the early relief of sore throat. This early symptomatic benefit may be partly related to the pharmacological characteristics of XFQOL, which contains 20 herbal ingredients. Several components have demonstrated antiviral, anti-inflammatory, and analgesic activities. For instance, Jinyinhua and Lianqiao are widely used in classical formulas for wind-heat exterior syndromes and have demonstrated inhibitory effects against respiratory viruses and modulation of pro-inflammatory cytokine responses (Qian et al., 2025; Yang et al., 2017). Peppermint, Great Burdock Achene, and Licorice Root have demonstrated analgesic, anti-inflammatory, anti-allergic, and throat-protective activities, which may contribute to the relief of sore throat, cough, and pharyngeal inflammation (Liu et al., 2013; Wahab et al., 2021; Jin et al., 2023). In addition, Gypsum Fibrosum and Paeoniae Radix Rubra in XFQOL are traditionally used to clear heat and cool the blood and have shown potential anti-inflammatory effects, which may partly explain the earlier improvement in sore throat and pharyngeal inflammation observed in the XFQOL group (Wang T. T. et al., 2024; Fan et al., 2024).

Despite the potential advantage in early sore throat relief, no statistically significant between-group differences were observed in the overall TCM syndrome response or disappearance rates, or in most individual symptoms and signs, including fever, cough with yellow sputum, pharyngeal and tonsillar signs, and lymphadenopathy. One possible explanation is that sore throat was the principal enrollment criterion, with a baseline WBS score of at least 4, thereby providing sufficient scope for detecting clinically meaningful improvement. In contrast, several other symptoms and signs were relatively mild at baseline and may therefore have had limited discriminatory capacity. It is also possible that XFQOL exerts more pronounced local throat-soothing and anti-inflammatory effects, whereas its effects on systemic manifestations are broadly comparable to those of the active control. Because no adjustment for multiplicity was applied to the secondary endpoint analyses, the corresponding P values should be regarded as nominal, and these findings should therefore be interpreted as supportive rather than confirmatory.

With regard to safety, XFQOL showed a favorable safety profile in this pediatric population. Only four adverse events were reported during the trial, all of which involved transient proteinuria and were assessed as possibly unrelated to the study medication. The incidence of adverse events did not differ significantly between the two groups. No serious adverse events, allergic reactions, or treatment-related adverse drug reactions were observed. These findings are consistent with previous studies of XFQOL in children with influenza or acute upper respiratory tract infections and further support its tolerability in children aged 3–14 years (Zhou et al., 2020; Guo et al., 2025).

However, this study had some limitations. First, WBS-assessed sore throat is a subjective participant-reported outcome, which may introduce measurement variability. Second, because there was no pure placebo group, the extent to which symptom improvement reflected drug-specific effects rather than spontaneous recovery in this self-limiting disease cannot be fully separated. Third, the sample size was modest and the trial was conducted at only three centers, which may limit the external validity of the results. In conclusion, XFQOL was non-inferior to the active control for pediatric acute pharyngitis/tonsillitis with wind-heat syndrome, showed favorable supportive and exploratory advantages in sore throat relief, and demonstrated a favorable safety profile.

## Data Availability

The raw data supporting the conclusions of this article will be made available by the authors, without undue reservation.
