# Correction: A multicenter, randomized, double-blind, double-simulated, positive drug parallel-controlled clinical trial to evaluate the efficacy and safety of Xiao’er Fengreqing oral liquid in the treatment of acute pharyngitis/ tonsillitis in children

**DOI:** 10.3389/fphar.2026.1988967

**Published:** 2026-10-09

**Authors:** Lei Shi, Kai Liu, Yike Song, Chunsun Mu, Chunxiang Wang, Yongchao Jiang, Robb Russell, Yaqian Wen, Rihe Liu, Jianping Liu, Huilan Liu, Zhaolan Liu

**Affiliations:** 1 Centre for Evidence-Based Chinese Medicine, Beijing University of Chinese Medicine, Beijing, China; 2 The Second Affiliated Hospital of Shandong University of Traditional Chinese Medicine, Jinan, Shandong, China; 3 Xiangyang Central Hospital, Xiangyang, Hubei, China; 4 Southern California University of Health Sciences, SCU Health, Whittier, CA, United States; 5 Center for Evidence-Based Medicine, Hospital of Chengdu University of Traditional Chinese Medicine, Chengdu, China; 6 Beijing University of Chinese Medicine Third Affiliated Hospital, Beijing, China

**Keywords:** acute pharyngitis, acute tonsillitis, multicenter, pediatrics, randomized controlled trial, traditional Chinese medicine, Xiao’er Fengreqing oral liquid

An incorrect Funding statement was provided. The correct funder is Central Government Guidance Fund for Local Science and Technology Development (264Z2501G). The correct funding statement reads:

The author(s) declared that financial support was received for this work and/or its publication. Backup Leader Support Program of Beijing University of Chinese Medicine (90010960920033); Double First-Class Construction Support Program - Key Discipline of Traditional Chinese Medicine Evidence-based Medicine of Beijing University of Chinese Medicine - Evidence-Based Medicine in Traditional Chinese Medicine (90010951310169); Central Government Guidance Fund for Local Science and Technology Development (264Z2501G).

The original version of this article has been updated.

